# Histomorphometric Assessment of Cancellous and Cortical Bone Material Distribution in the Proximal Humerus of Normal and Osteoporotic Individuals: Significantly Reduced Bone Stock in the Metaphyseal and Subcapital Regions of Osteoporotic Individuals

**DOI:** 10.1097/MD.0000000000002043

**Published:** 2015-12-28

**Authors:** Christoph M. Sprecher, Florian Schmidutz, Tobias Helfen, R. Geoff Richards, Michael Blauth, Stefan Milz

**Affiliations:** From the AO Research Institute Davos, Davos, Switzerland (CMS, FS, TH, RGR, SM); Department of Anatomy (CMS, SM); Department of Orthopaedic Surgery, University of Munich (LMU) (FS); Department of General-, Trauma-, Hand and Plastic Surgery, University of Munich (LMU), Munich, Germany (TH); and Department of Trauma Surgery, Medical University of Innsbruck, Innsbruck, Austria (MB).

## Abstract

Osteoporosis is a systemic disorder predominantly affecting postmenopausal women but also men at an advanced age. Both genders may suffer from low-energy fractures of, for example, the proximal humerus when reduction of the bone stock or/and quality has occurred.

The aim of the current study was to compare the amount of bone in typical fracture zones of the proximal humerus in osteoporotic and non-osteoporotic individuals.

The amount of bone in the proximal humerus was determined histomorphometrically in frontal plane sections. The donor bones were allocated to normal and osteoporotic groups using the T-score from distal radius DXA measurements of the same extremities. The T-score evaluation was done according to WHO criteria. Regional thickness of the subchondral plate and the metaphyseal cortical bone were measured using interactive image analysis.

At all measured locations the amount of cancellous bone was significantly lower in individuals from the osteoporotic group compared to the non-osteoporotic one. The osteoporotic group showed more significant differences between regions of the same bone than the non-osteoporotic group. In both groups the subchondral cancellous bone and the subchondral plate were least affected by bone loss. In contrast, the medial metaphyseal region in the osteoporotic group exhibited higher bone loss in comparison to the lateral side.

This observation may explain prevailing fracture patterns, which frequently involve compression fractures and certainly has an influence on the stability of implants placed in this medial region. It should be considered when planning the anchoring of osteosynthesis materials in osteoporotic patients with fractures of the proximal humerus.

## INTRODUCTION

Osteoporosis is a systemic skeletal disorder, which causes reduction of the bone stock or/and quality and impairs biomechanical stability of the skeleton.^1^ It affects predominantly postmenopausal women but also occurs in men at an advanced age.^2^ Proximal humerus fractures are among the 4 most frequent types of fractures in the elderly population (i.e. aged ≥65 years) and may already occur after minor trauma.^3^ These fractures still pose a challenge for adequate stabilization in modern osteosynthesis.^4^ Despite all advances in the field of osteosynthesis material development there are still considerable problems related to the occurrence of screw cut-out phenomena as well as short- and long-term implant instability.^4,5^

This is underlined in a previous study^4^ involving 53 elderly patients (mean age 63 years, 72% females) who had proximal humeral fracture, which was treated with an angular stable plate. Primary screw perforation during the operation was the most frequent problem with 13.5% followed by secondary screw perforation with 7.3%. In a recently published prospective multicenter study (131 patients, mean age 66 years, 70% females) involving a polyaxial angular stable plate the most frequent implant-related problem was intra-articular screw perforation occurring in 14.5% of patients.^6,7^ Several other studies have resulted in comparable outcomes.^8–14^

The current clinical picture indicates a particular problem related to the fact that no surgically accepted “bone material distribution map” of the proximal humerus exists to give a good forecast for potentially useful implant anchoring positions. As a result the stable placement of implants can be very difficult, especially in the case of an osteoporotic fracture.

A particular problem in all studies comparing normal and osteoporotic individuals relates to the fact that there are no generally accepted rules for their classification. This question has been addressed in several studies and led to a recommendation by the WHO to classify normal and osteoporotic conditions using the T-score.^15^ Currently, the authors follow the WHO classification and differentiate between normal and osteoporotic individuals based on T-scores obtained by DXA measurements of the distal radius.

It is already known that osteoporosis does not affect all regions of the upper skeleton to the same extent^16,17^ and thus it cannot be assumed that reduction of the bone stock or/and quality occurs more or less homogenous in all parts of a larger human bone like the humerus.

Therefore, the aim of the present study was to investigate the distribution of bone tissue within the proximal end of the humerus in frontal sections of normal and osteoporotic human samples. Due to physiological differences in the regional bone structure and material distribution, we compared different regions of cancellous and cortical bone in the proximal humerus and defined the regions with respect to the occurrence of typical fracture lines^18^ in an elderly patient collective. For cancellous bone we choose the bone volume to total volume (BV/TV) ratio as an appropriate parameter for assessment of material distribution (bone density) whereas in the case of compact bone, we used the cortical or subchondral plate thickness as representative parameters.^19–21^

## METHODS

### Donors

Upper extremities including the shoulder joint from 12 donors (average age 68.6 years, age range: 19–90 years, 6 males, 6 females; further details are given in Table 1) were obtained from Platinum Medical (Herderson, NV). Specimens were fresh frozen and had been collected postmortem with appropriate consent of the individual or of their relatives. The specimens were handled according to legal regulations of Switzerland.

**TABLE 1:**
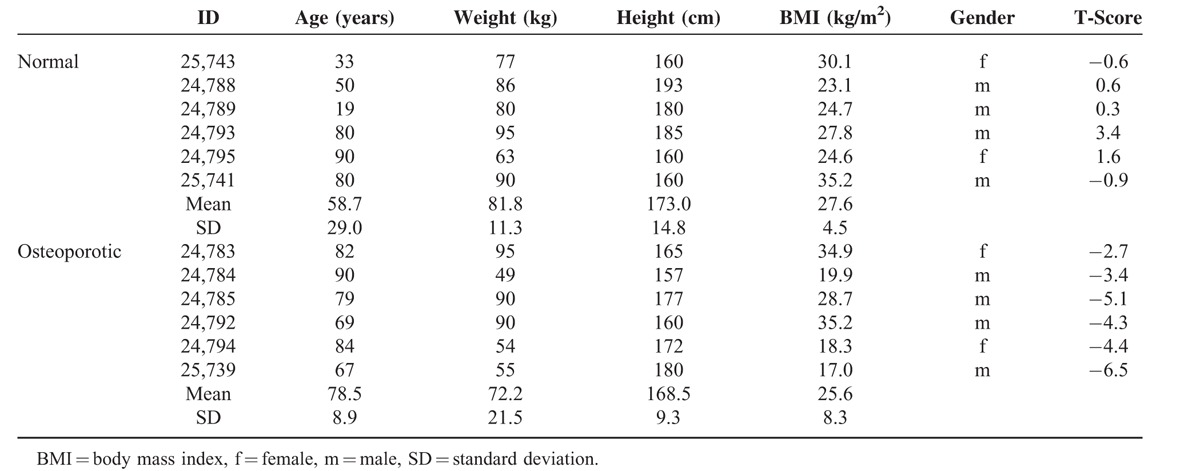
Individual Donor Data Presentation

DXA measurements from the distal radius, ipsilateral to the proximal humerus used for histomorphometry, were obtained for each specimen using a DXA scanner (GE Healthcare Lunar Prodigy DF+14868, Madison, WI) and the T-score was recorded as recommended by the WHO. Donors were grouped into normal and osteoporotic individuals using the T-score as a criterion for decision (details in Table 1). This approach seemed reasonable because Krappinger et al.^14^ could demonstrate a correlation (correlation coefficient 0.57) between the average bone mineral density (BMD) values of the radius and humeral head in living human patients.

### Specimen Preparation

After thawing, the specimens were dissected and the proximal third of the humerus was removed and fixed for at least 4 weeks in 70% methanol and then were dehydrated in ascending concentrations of alcohol at room temperature. Finally, the proximal humeral end was block embedded in methylmethacrylate and polymerized in a temperature controlled water bath.^20^After hardening of the block, 1 section per specimen was obtained in the frontal plane with a diamond band saw (Exakt Makro Diamond Band Saw, Norderstedt, Germany). Each section with a thickness of ∼500 μm was glued on a custom made plastic slide (size 55 × 110 mm), ground and polished with an Exakt grinding 400CS (EXAKT, Norderstedt, Germany) to a thickness of ∼400 μm and finally stained with Giemsa Eosin stain.

For overview images the stained sections were scanned with an Umax Powerlook Scanner (Umax 2100XL). Detailed images at higher resolutions at selected locations within the sections were made using a Zeiss Axioplan microscope (Zeiss, Göttingen, Germany) equipped with a high resolution camera (Axiocam HRc).

### Definition of the Regions of Interest for Cancellous Bone Material Distribution Assessment

**FIGURE 1:**
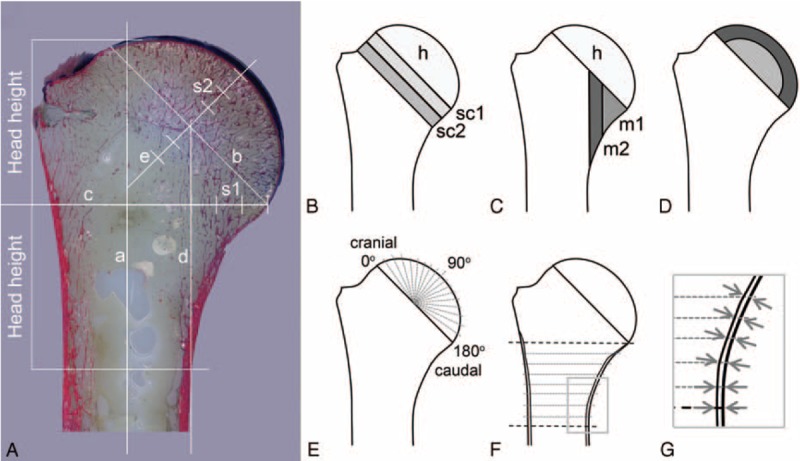
Schematic diagram demonstrating the different regions assessed in all humeri. (A) Giemsa Eosin stained section with geometric overlay showing all lines and distances used for definition of the regions of interest and locations of the measuring points. (B) Sketch drawing with the cancellous regions of the humeral head (h), and the 2 subcapital regions (sc1, sc2). (C) Sketch drawing showing the metaphyseal regions m1 (medial region) and m2 (lateral region). (D) Outer subchondral (dark gray) and inner (light gray) cancellous regions of the humeral head. (E) Sketch drawing showing the location of the measuring points where the subchondral plate thickness was measured. The humeral head joint surface forms a semicircle and the head center is used to cover with measuring points every 10 degrees of rotation. (F) The cortical thickness was assessed in 8 regular intervals medially and laterally (for details of the measurements, see also G).

The histological section of the proximal end of the humerus was separated into different regions of interest and these regions then were morphometrically assessed. To achieve an unbiased and reproducible determination of the boundaries of the various regions in all the humeri, the following geometric scheme was applied. First, the central long axis of the humerus was determined (line a in Figure 1A) then line b was drawn as the connection between the cranial and caudal end of the hyaline articular cartilage covering the head. This line was considered as a reproducible identifier for the course of the “collum anatomicum” or anatomical neck. Further, a line c, perpendicular to the long axis of the humerus (line a), was constructed in a way that it met the caudal end of line b at the point where the cartilage ended. This line was divided into a medial and a lateral segment by line d, which was parallel to the long humeral axis (line a) and covered the periosteal segment at the distal medial end of the proximal humerus. The medial segment of line c was divided into 3 segments of equal length (s1 in Figure 1A) which were used later to define the long boundaries of the 2 medial metaphyseal regions m1 and m2 as shown in Figure 1C.

The regions of the humeral head were defined through a line e, which ran through the central point of line b and perpendicular through it. Line e ended at the beginning of the subchondral plate, which was not included into the bone density assessment and was divided into 3 segments (s2 in Figure 1A) of the same length. The length of s2 was used to construct the 2 subcapital regions sc1 and sc2 (Figure 1B). Both regions did not include the cortical bone lamellae at either end. The rest of the cancellous bone next to region h (head without subchondral plate) represented the bone stock of the humeral head (Figure 1B and C). In a further step it was divided into an inner and subchondral region (Figure 1D), using again the length of s2 as an unbiased geometric parameter for topographical separation of the regions.

### Definition of the Regions of Interest for Cortical Bone and Subchondral Plate Thickness Assessment

The previously defined geometric parameters were used as landmarks for definition of the points where the thickness of the subchondral plate was measured. The latter was defined as the distance from the end of the cartilage, stained in deep blue in the Giemsa Eosin stained sections, to the beginning of the marrow cavity (unstained). Measurements were obtained at intervals of 10 degree using the central point of line b as the centre of the semicircle representing the humeral head (Figure 1E).

The cortical thickness of the medial and lateral compact bone lamella was obtained at 9 points on each side of the humerus. In order to assess comparable skeletal regions in different individuals and to account for the individual geometry of the bones the position of these points was defined using the height of the humeral head as the reference distance, which was divided into 8 segments of equal length. Starting at the level of line c (Figure 1A) 9 medial and lateral cortical thickness values were obtained (Figure 1F and 1G).

### Histomorphometry and Statistical Evaluation

Histomorphometric image analysis was performed with the aid of KS400 Image analysis software (Zeiss, Göttingen, Germany). Trabecular bone volume (BV/TV)^19,21^ as a surrogate measure for cancellous bone material distribution (bone density), cortical bone, and subchondral plate thickness^20^ as a measure for compact bone distribution were determined interactively on the Giemsa Eosin stained sections using custom-made KS400 macros.

Results were statistically evaluated using SPSS version 21 (IBM SPPS, Armonk, NY). For detection of normally distributed values the Shapiro–Wilk Test was used. Regional values were compared using the General Linear Model Repeated Measures or the Related-Samples Wilcoxon Signed Rank Test with Bonferroni correction.

Comparisons between the 2 groups were performed using t-test for normally distributed values and Related-Samples Wilcoxon Signed Rank Test for non-normally distributed values. Significance level was set at *P* = 0.05 for all statistical tests.

## RESULTS

### Groups and Samples

The average age of the 6 donors from the normal (nonosteoporotic) group was 59 years (± 29 years standard deviation, range 19–90) and 79 years (± 9 years standard deviation, range 67–90) for the 6 donors of the osteoporotic group. In the normal group the T-score obtained from DXA measurements at the distal radius of the same arm ranged from −0.9 to 1.6 and in the osteoporotic group from −2.7 to −6.5 (Table 1). No information regarding the dominant extremity of the donor was available.

### Histomorphometry of the Cancellous Bone of the Humerus

#### Subcapital Region

**FIGURE 2:**
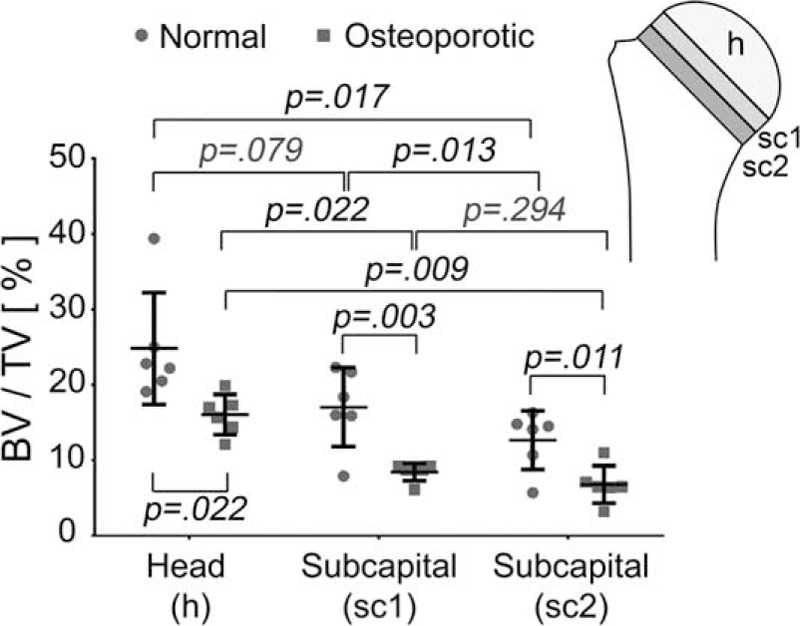
Comparison of the histomorphometrically determined bone density (BV/TV) in different regions (h = head, sc1 = subcapital region 1, sc2 = subcapital region 2) of the normal and osteoporotic group. In all regions the bone density was significantly lower in the osteoporotic group when compared with the normal group. Plots indicate average values with standard deviation. BV/TV = bone volume to total volume.

**FIGURE 3:**
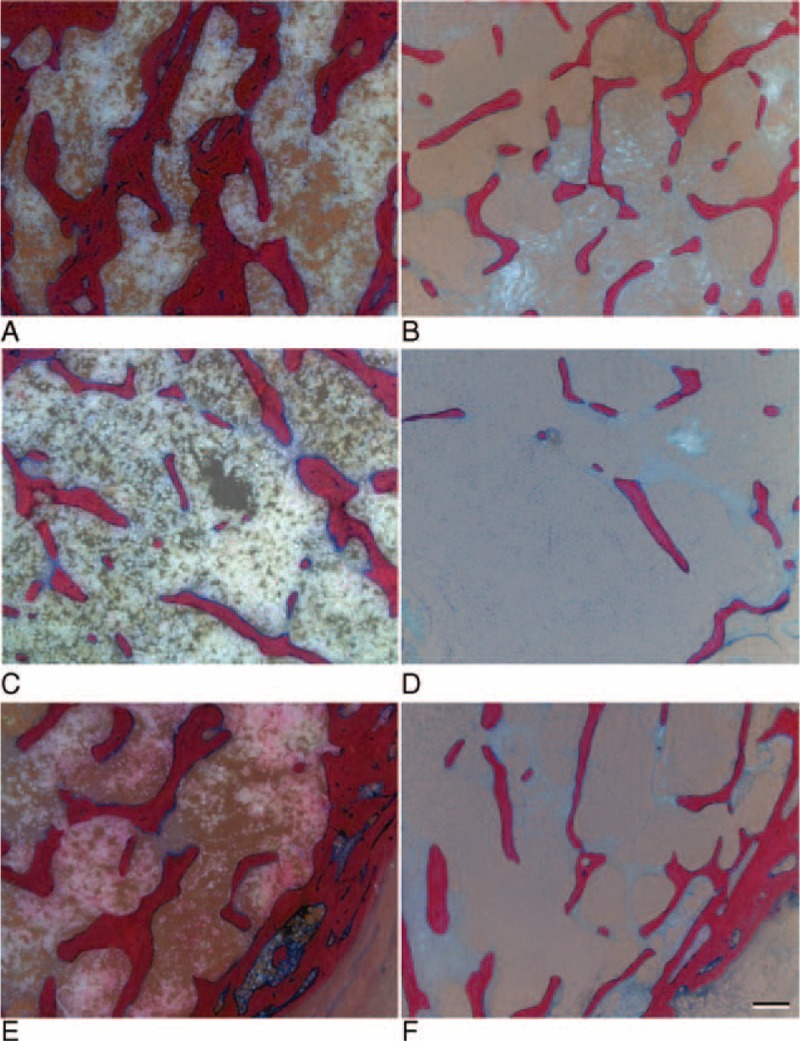
Examples of the morphology of cancellous bone in normal bone (A, C, E) and osteoporotic bone (B, D, F) obtained from Giemsa Eosin stained thick methylmethacrylate sections. The regions in the middle of the head are compared in A and B, and the subcapital regions in C and D. In E and F the region at the medial metaphysis is shown and the cortical bone is visible in the lower left corner of each image. (Scale bar 500 μm).

The apparent density of the cancellous bone varied between the different subcapital regions of the humeral head in normal and osteoporotic donors (Figures 2 and 3). The cancellous region of the humeral head, which did not include the subchondral plate, showed the highest bone density values. The values were significantly reduced in the subcapital regions near the “collum anatomicum”. All regions investigated exhibited a significant decrease of bone density in the osteoporotic group when compared to the normal group (Figures 2 and 3). The most significant difference between the values from the osteoporotic and the normal group was found in the first third of the subcapital region (region labeled sc1 in Figure 2); here the reduction of bone density was most pronounced.

#### Metaphyseal Region

**FIGURE 4:**
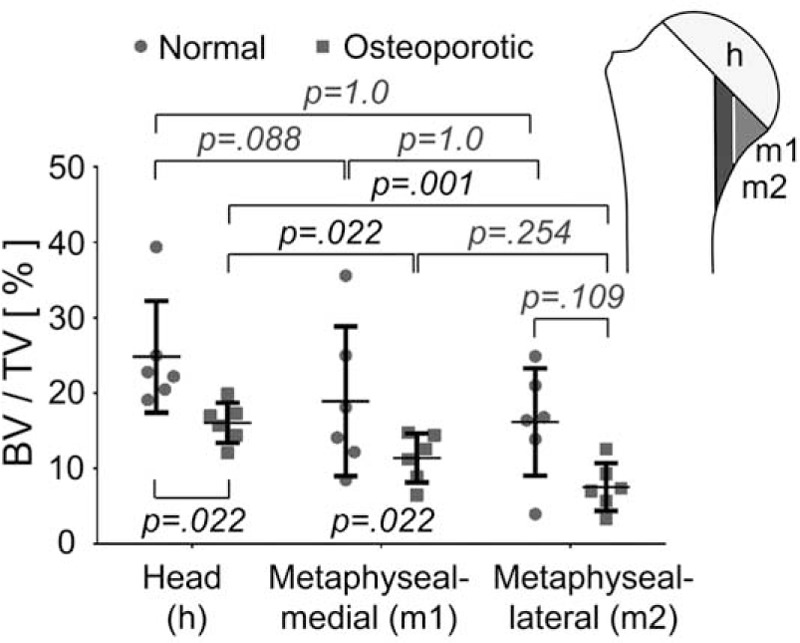
Comparison of bone density (BV/TV) between the normal and osteoporotic groups in the head and 2 regions on the medial side of the metaphysis. Significant differences between the regions were found only in the osteoporotic group. Plots indicate average values with standard deviation. BV/TV = bone volume to total volume.

When the bone density of the humeral head was compared to regions at the medial side of the metaphysis, the 3 regions showed no significant differences in the normal group but in the osteoporotic group significant reduction of bone density occurred in the 2 regions of the metaphysis (Figures 3 and 4).

#### Subchondral Region

**FIGURE 5:**
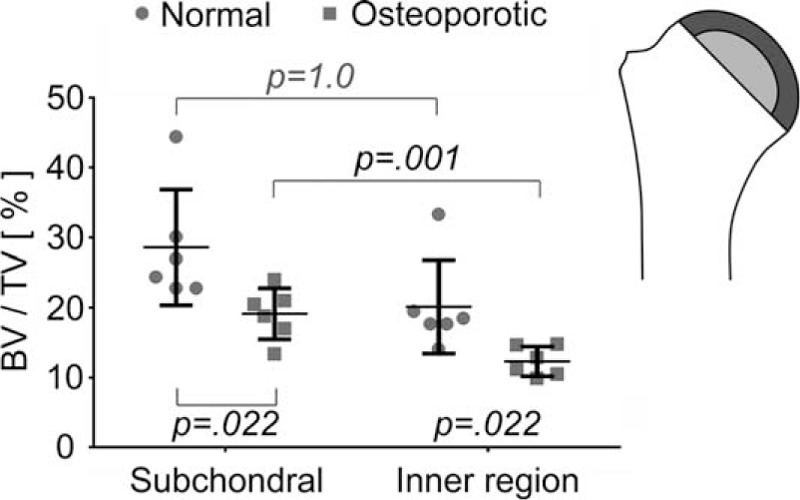
Humeral head bone density (BV/TV) of the normal and osteoporotic group in the subchondral (dark gray) and the inner region (light gray) has shown significant differences in the osteoporotic but not in the normal group. The bone density of the osteoporotic group in both regions is significantly lower than that in the normal group. Plots indicate average values with standard deviation. BV/TV = bone volume to total volume.

As the region of the humeral head was relatively large compared to the other regions, it was decided to divide the head in 2 regions, 1 of them closer to the subchondral plate than the other. The bone density values in these 2 regions showed no difference for the normal group but a highly significant difference for the osteoporotic group. Both regions showed a significant bone density reduction in the osteoporotic group when compared to the normal group (Figure 5).

### Cortical Dimensions of the Proximal Humerus

#### Thickness of the Subchondral Plate

**FIGURE 6:**
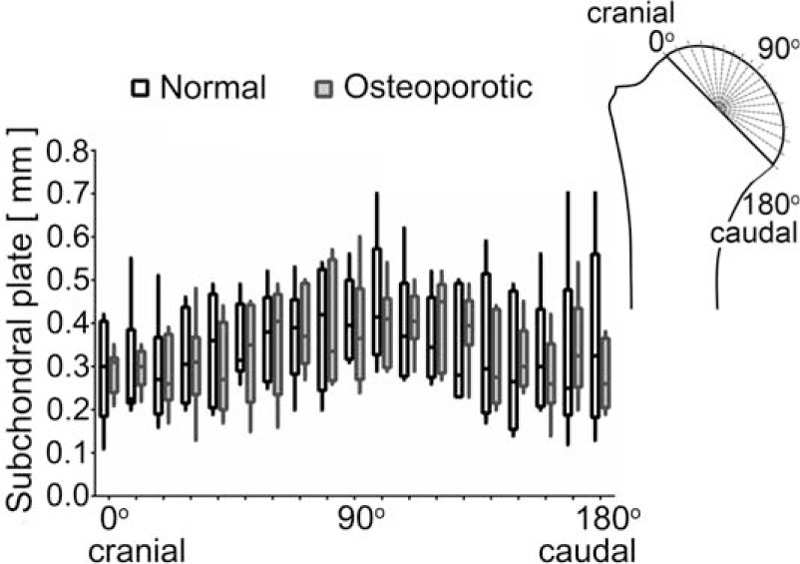
The thickness of the subchondral plate did not show any significant differences between the normal and osteoporotic group or between the different locations.

**FIGURE 7:**
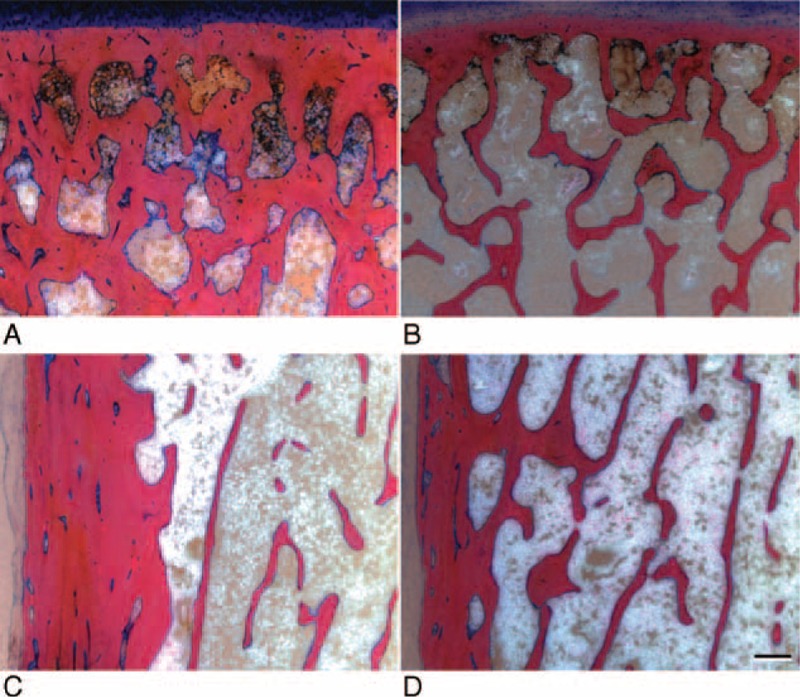
Examples of compact bone morphology in normal bone (A, C) and osteoporotic bone (B, D). A and B show a decrease of subchondral plate thickness and C and D show the decrease of cortical bone thickness in the lateral metaphyseal region. (Scale bar 500 μm).

The thickness of the subchondral plate supporting the articular cartilage was measured at defined locations in both groups, but revealed no statistically significant differences between the osteoporotic and normal group or the different locations within both groups (Figures 6 and 7).

#### Thickness of the Metaphyseal Cortex

**FIGURE 8:**
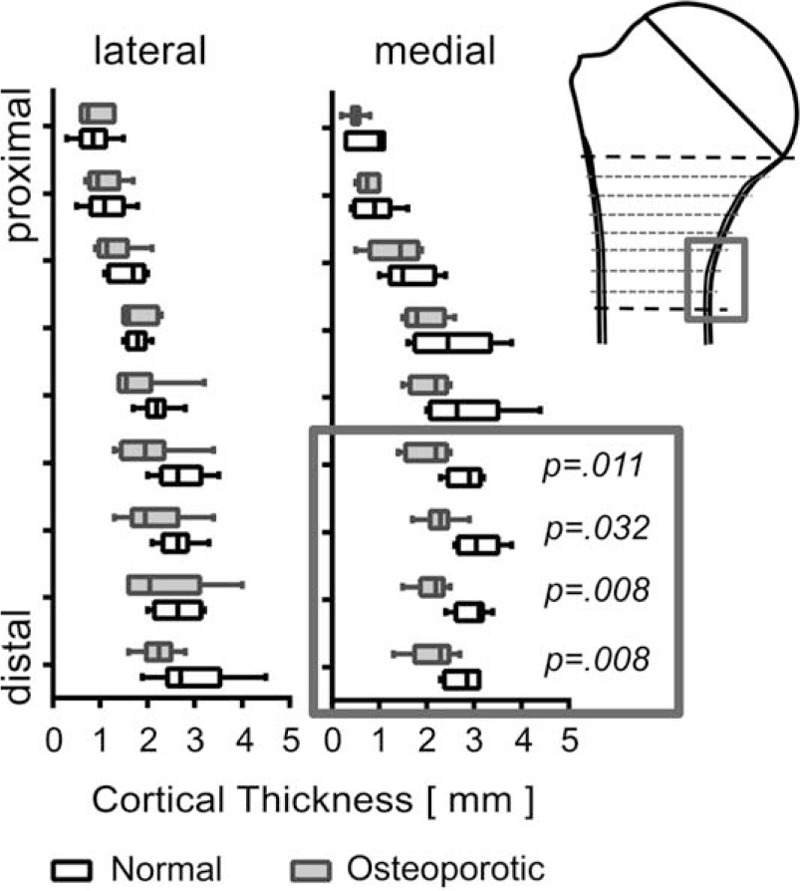
The investigation of cortical thickness at the lateral and medial sides of the metaphyseal region exhibited considerable differences between the different locations in both groups. Only for the most distal locations of the medial cortex (highlighted by dark gray boxes) the thickness values from the normal and osteoporotic group showed significant differences.

The thickness of the cortical wall was measured medially and laterally at 9 points each. Only on the medial side the 4 most distal measuring sites exhibited significant differences between the 2 groups (Figures 7 and 8).

## DISCUSSION

Osteoporosis is seen as a systemic condition, which affects the bone metabolism of the entire body.^1^ As such it is often assumed that the bone stock and/or quality reduction process is more or less equally affecting all regions of the skeleton. Our results demonstrate that this is not the case in the human proximal humerus and that certain topographical regions are more prevalent to bone reduction than others. Comparable findings have been reported for the human distal humerus,^16^ distal radius,^17^ and for the proximal femur.^22–24^ The fact that bone material reduction occurs in a nonuniform way in different regions with cancellous bone has implications for the fracture risk potential and subsequent treatment of osteoporotic humeral head fractures and our results may also help to predict regions in osteoporotic humeri, which are likely more suitable for anchoring of osteosynthesis materials in cases of fracture than others.

Our results also show that the humeri of normal individuals exhibit significant regional cancellous bone density variations and that these distribution patterns are changed under osteoporotic conditions.

The cancellous bone of the humeral head had the highest bone density in regions close to the subchondral plate. Closer to the anatomical neck the bone material density decreased and this effect was becoming more and more pronounced in osteoporosis. The regionally distinct and increasing degree of osteoporotic cancellous bone reduction is best reflected by the significant bone material decrease in the 2 medial metaphyseal regions. These 2 regions exhibit more bone loss than the corresponding osteoporotic humeral head, which also shows significant bone reduction when compared to a normal humeral head. It is interesting to note that a significant difference between the bone density values of the head and these 2 regions were only observed in the osteoporotic group whereas no differences were detected in the normal group. It is worth noting that in osteoporotic patients this region often fails to withstand the compressive stresses acting on the typically 1 superiorly placed fragment of the fractured head and that this caused deterioration of the stability of a surgically treated humeral head.^25^ It is also worth to note that this region is already showing a tendency towards lower bone density values in normal patients.

In normal patients the humeral head has a relatively uniform cancellous bone density, which is significantly reduced in the central and especially subcapital regions in the osteoporotic group. The osteoporotic bone reduction process obviously affects certain regions more severe than others among them especially the cancellous bone at the level of the anatomical neck. This weakening of the humeral head stability is well reflected by the characteristic shape of frequently occurring humeral head fragments in osteoporotic patients.^18^

In the same context it was of interest to check whether the subchondral bone plate, which consists of the subchondral bone and the overlying mineralized cartilage, was reduced in thickness under osteoporotic conditions. This clearly was not the case in our investigation and it looks as if the humeral subchondral plate thickness is a parameter which is not or much less affected by osteoporosis. At present we can only speculate on the reasons for this observation. It has however been observed at other locations of the skeleton that cancellous bone may be much earlier affected by osteoporosis than cortical bone^26^ and it could well be that there is another difference for the subchondral plate beneath an articular joint and the shaft cortical bone. Here it has to be noted that the subchondral plate is consisting not only of bone but also of mineralized cartilage^20^ and that the composition may vary considerably between individual joints.

Since the stability of the shaft of a long bone is well determined by the thickness of the cortical bone, it was most interesting to see how the transition zone, where the load-bearing function is shifted from the cancellous bone of the humeral head to the shafts cortical bone, would be altered in osteoporosis. Interestingly, the only significant difference we could determine was seen in the medial and distal cortical bone covering the medial metaphyseal regions. This was surprising because in the femoral neck region Zebaze & Seemann^27^ could demonstrate significant changes of cortical thickness between normal and osteoporotic individuals. As mentioned before, in proximal humerus fractures the mechanical properties of the medial metaphyseal region are of paramount importance for the stability of a locked Plate^25^ or a intramedullary nail osteosynthesis.^28^ All other measuring points and this means also the entire lateral side of the humerus did not show a significant difference in cortical bone thickness when osteoporotic and normal humeri were compared.

Although our investigation is only using the humeri and radii from 12 donors, subdivided into 2 equally sized groups of normal and osteoporotic individuals, we could observe significant differences in bone material distribution and cortical thickness in various regions of the proximal humerus. This however is only achievable because the histomorphometric determination of bone material distribution (i.e. bone area per field of view) was made in large resin embedded sections of undecalcified bone which allows for high imaging resolution. The latter is mandatory for the reliable determination of thin bone structures. These thin cancellous or cortical structures cannot be reliably detected with other methods such as μ-CT or clinical CT because the current voxel sizes coincide with partial volume effects which affect predominantly regions with very low bone density and few fine structures. It however can be argued that we only investigated 1 section and not the entire volume of a proximal humerus. This is owed to the complexity of the measuring process and the geometrical determination of the regions and points of interest. Since all proximal humeri are of different size and shape, our approach aims to standardize the choice of randomly selected regions of interest. Using reproducibly determined regions of interest for morphometric bone, material distribution assessment is an important advantage of our study design. Moreover, it is a necessary precondition for the statistical analyses we performed.

Clinically, our results render the medial metaphyseal region as not very sufficient for implant (i.e. screw) anchoring in osteoporotic patients. In these patients it would probably be more successful to use longer screws aiming at regions where more bone stock is present.

However, our results are based on single sections in the frontal plane of proximal humeri obtained from a limited number of donors. Thus the surgeon has to consider other out-of-plane regions which may potentially provide sufficient implant anchoring capacity.

Our results show that the various regions of the proximal humerus exhibit different bone material distributions in normal and osteoporotic individuals. Osteoporotic individuals show more pronounced differences than normal individuals in various regions of the proximal humerus, inhomogeneously affected by bone loss. Especially the medial metaphyseal region experiences a particularly high bone loss and thus biomechanical weakening. This may influence the prevailing fracture patterns and also interferes with osteosynthesis stability.
